# Dr. Martin S. Kittinger

**Published:** 1904-10

**Authors:** 


### OBITUARY.

Dr. Martin S. Kittinger, of Lockport, died at his home in that city, September 11, 1904, aged 77 years. He had been in declining health since last winter when he took cold, which clung to him with tenacious severity. Nevertheless, he kept at his professional work, even going into the country during the severest weather in response to professional calls. In June he visited Atlantic City, but was not benefited by the change. He, however, continued his work after his return, making a stubborn contest with the disease that his rugged constitution resisted for a time, but could not conquer.

Dr. Kittinger was a native of Niagara County in which he lived all his life, excepting during four years devoted to army service. He graduated in medicine at the College of Physicians and Surgeons in 1853, and began practice in his native county. He was commissioned surgeon of the 100th Regiment, New York Volunteers, February 2, 1862, and served in the Army of Potomac until mustered out, January 9, 1865. He acquired fame as an army surgeon, and was put in charge of a field hospital of division, his rank being that of major.

After leaving the military service he returned to Lockport, where he soon built up a large practice and became a leading surgeon in the county. He also was accomplished as an ophthalmologist.

Dr. Kittinger was one of the organisers of the Lockport Business Men's Association and at his death one of its directors. He was a member of the Medical Society of the County of Niagara and for years its president. He was twice married, his first wife being Laura M. Day, who died about thirty-four years ago. She left him three sons, who survive the father. They are: Martin S. Kittinger, Jr., Harry D. Kittinger and Dr. Ferdinand A. Kittinger of Lockport, coroner of Niagara County. His present wife was Emma Lackor, who bore him one daughter, Mrs. Seth Van Loan, of Philadelphia, who has spent the summer at home at her father's bedside.

Dr. Kittinger was a man of strong personality, a striking figure, with keen eyes and raven black hair, showing not a trace of gray. He will be sadly missed by his family and in the community in which he bore a prominent part as a sterling citizen.

